# How implicit social information shapes spatial memory of objects

**DOI:** 10.1007/s00426-026-02254-0

**Published:** 2026-02-24

**Authors:** Scila Nunziata, Tina Iachini, Gennaro Ruggiero

**Affiliations:** Department of Psychology, Laboratory of Cognitive Science and Immersive Virtual Reality, CS-IVR, University of Campania L. Vanvitelli, Viale Ellittico, 31, Caserta, 81100 Italy

**Keywords:** Interpersonal distances, Spatial reference frames, Spatial memory, Social cognition, Mutual gaze

## Abstract

In our environments, we encode surrounding spatial information using egocentric (subject-to-object) and allocentric (object-to-object) reference frames. Spatial encoding, however, occurs in an environment populated by more than just “objects” but also by people, and this social information can have a significant impact on our spatial memory. Here, we investigated how implicit social information influences spatial encoding by designing a study with an explicit spatial task and implicit social cues. Participants performed a task where they memorized triads of geometric objects and provided egocentric and allocentric judgments of relative distance. Each object was positioned in front of pairs of social (virtual humans) and non-social stimuli (lamps and chairs, as control conditions). These stimuli, irrelevant to the spatial task, could be at different proxemic distances (intimate, personal, and social), and with mutual and non-mutual gaze (Facing/Not-Facing), in the case of virtual humans and chairs. A questionnaire assessing empathic disposition was also administered. The overall pattern of results showed that the egocentric processing was facilitated over the allocentric one when the social cues were clear and allowed an easy social categorization. Notably, no such advantage emerged when social categorizations required further processing to understand social relations. The empathic disposition was also associated with the spatial performance. In conclusion, our findings demonstrate that even when irrelevant to a task, social information, defined by nonverbal signals (proxemics distance and gaze), implicitly affects the way we represent our surrounding environment. This highlights the intertwined nature of spatial cognition and social processes in everyday life.

## Introduction

Imagine visiting a museum. While walking through the halls to admire paintings and sculptures, you encounter other visitors (e.g., friends or unknown people), who stand near or far from each other and exchange gazes (non-verbal signals). This simple example shows how in encoding spatial information (i.e., the position of paintings) we are, at the same time, exposed to social information (i.e., people around us). Here, we wondered if social information can implicitly influence how we represent surrounding spatial information.

In the literature, this topic draws on two research traditions: spatial cognition, which studies how the positions of stimuli in space (e.g. objects, landmarks) are represented; and social psychology, which studies how social information is processed.

Regarding the spatial domain, research has shown that the spatial structure of the physical environment can be represented through egocentric (subject-object) and allocentric (object-object) spatial reference frames (Iachini, 2024; Ruggiero et al., 2016; Hu et al., 2018; Millar, 1994). Generally, the egocentric encoding shows an advantage over the allocentric one, as it is based on immediate bodily references, which are easier to process and less cognitively demanding (Iachini et al., 2015). In contrast, allocentric encoding requires more complex transformations and the integration of spatial relations between stimuli, thus being more challenging (Ruotolo et al., 2015; Hu et al., 2018; Millar, 1994).

Neuroimaging studies have shown the involvement of a posterior parieto-frontal premotor network in egocentric encodings, and of medio-temporal and parieto-temporal regions in allocentric encodings (Antonova et al., 2009; Committeri et al., 2004; Derbie et al., 2021a, b; Moffat et al., 2006; Parslow et al., 2004; Ruotolo et al., 2019; Schindler & Bartels, 2013; Vallar et al., 1999; Zaehle et al., 2007). However, individuals do not live in a purely physical space. We share the environment with other people and automatically map their social relationships, even in the absence of direct social interactions (de Vignemont, 2008). For example, while travelling on the metro, one may observe two passengers standing close to each other. Based solely on their spatial proximity and body orientation, the observer may infer whether or not these two individuals know each other.

Regarding social psychology, categorization is fundamental to human cognition as it organizes the knowledge about our conspecifics in meaningful ways (e.g., ethnicity, familiar/unfamiliar group, membership and so forth; Frith & Frith, 2007; Dovidio et al., 1989; Turner & Tajfel, 1979; Hogg & Turner et al., 1987). Social categorization facilitates inferences about others’ intentions and behaviors through the identification of similarities and differences (e.g., Smith & Medin, 1981; Bodenhausen et al., 2012; Fiske & Macrae, 2012; Frith, 2008; McGarty et al., 2015). For example, when individuals encounter unusual category combinations (e.g., a female bricklayer), they automatically engage in cognitively effortful explanatory processing (Hutter & Wood, 2015). This is in line with social psychology models explaining that social perception begins in a stereotypical, category-based manner but becomes more analytical and complex when existing categories do not easily fit the person being perceived (Fiske & Neuberg, 1990). Alongside, research in nonverbal communication has examined how interpersonal distance and gaze behavior, two key components of proxemics, mediate the categorization of others (Hall, 1966; Hayduk, 1983; Mehrabian & Wiener, 1967). Proxemics investigates the interpersonal distance that individuals maintain from each other to ensure a sufficient level of comfort and social appropriateness (e.g., Hall, 1966; Hayduk, 1983; Mahrebian & Wiener, 1967; Iachini et al., 2014a, b; Fiske, 1992). Hall (1973) divided the interpersonal distances into Intimate (0–45 cm; e.g., close relations), Personal (45–120 cm; e.g., conversations with friends or formal relationships), Social (120–300 cm; e.g., interactions with unfamiliar individuals), and public (300–500 cm: e.g., public gatherings). Similarly, mutual gaze, as deictic signal in nonverbal human communication, regulates the degree of comfort of a social interaction. For example, comfortable distances promote mutual gaze, and uncomfortable distances reduce it (Argyle & Dean, 1965; Argyle & Graham, 1976; Mendelson et al., 1982; Hayduk, 1978; Kendon, 1969; McCall, 2017; Patterson, 1977). Gaze represents a powerful communicative cue, as it not only conveys meaningful information (Kleinke, 1986; Driver et al., 1999; Batki et al., 2000; Emery, 2000; Frith, 2008) but also contributes to inferential processes that they may play a role in the interpersonal dynamics of others (Allison et al., 2000; Pierno et al., 2008).

Overall, interpersonal distance and gaze activate social categorization processes that enable us to understand and predict the mental states, intentions and actions of others, and to modify our own behaviour accordingly (Frith & Frith, 2007; McGarty et al., 2015). Consistently, people automatically generate representations of complex social constructs, such as intimacy, personal and public relationships, and interpersonal attitudes, based on distances and orientations (McCall, 2017).

A growing line of research is demonstrating a close link between social and spatial information to the point of proposing a social spatial cognitive map (Dorfman et al., 2021; Eichenbaum, 2017; Tavares et al., 2015). For example, Peer et al. (2021) demonstrated that the structure of real-world social networks are encoded in brain regions traditionally associated with spatial cognition (e.g., retrosplenial cortex). Consistently, evidence has shown that the presence of conspecifics shapes spatial behaviour, i.e., the way spatial environment is explored and represented (for animals see Dorfman et al., 2021), spatial orientation (Gobel et al., 2018), perspective taking (e.g., Galati & Avraamides, 2015; Gunalp et al., 2019; Tversky & Hard, 2009; see also Chen & McNamara, 2011), and spatial distance (Maddox et al., 2008; Nunziata et al., 2023). For example, Gunalp et al. (2019), through a spatial perspective-taking task, found a better performance when the scene included a virtual human or a chair (familiar element), compared to abstract directional cues like arrows. Importantly, Maddox and colleagues (2008) asked participants to estimate the spatial distance of stores located in different neighbourhoods. The store owners belonged to different ethnicities (social factor). They found that stores managed by people of the same ethnicity were perceived as closer as compared to those of different ethnicity. Overall, this evidence suggests that social information (avatar/chair) and social categories (ethnicity) were not only implicitly incorporated into spatial encoding but even influenced it. Consistently, recent models of social cognition have proposed that brain constructs ‘social cognitive maps’ analogous to spatial maps, representing relationships between people through both reference systems: egocentric, oriented toward self-other interaction, and allocentric, oriented towards knowledge of context and other-other interaction (de Vignemont, 2008; Frith and de Vignemont, 2005; Arzy & Kaplan, 2022; Eichenbaum, 2017; Tavares et al., 2015).

### Overview of the study

In sum, previous literature suggests a close link between social processing and spatial processing. Indeed, social information, such as social category (Maddox et al., 2008), has been shown to affect spatial behaviour. Similarly, neurofunctional evidence (e.g., Tavares et al., 2015; Peer et al., 2021) has shown that neural systems traditionally associated with spatial cognition were also associated with the representation of the social world. However, it is unclear if and how social cues available in the environment can implicitly influence the spatial representation of stimuli. Furthermore, it is not clear whether they exert a similar or different influence on the encoding of egocentric and allocentric spatial reference frames.

**Fig. 1:**
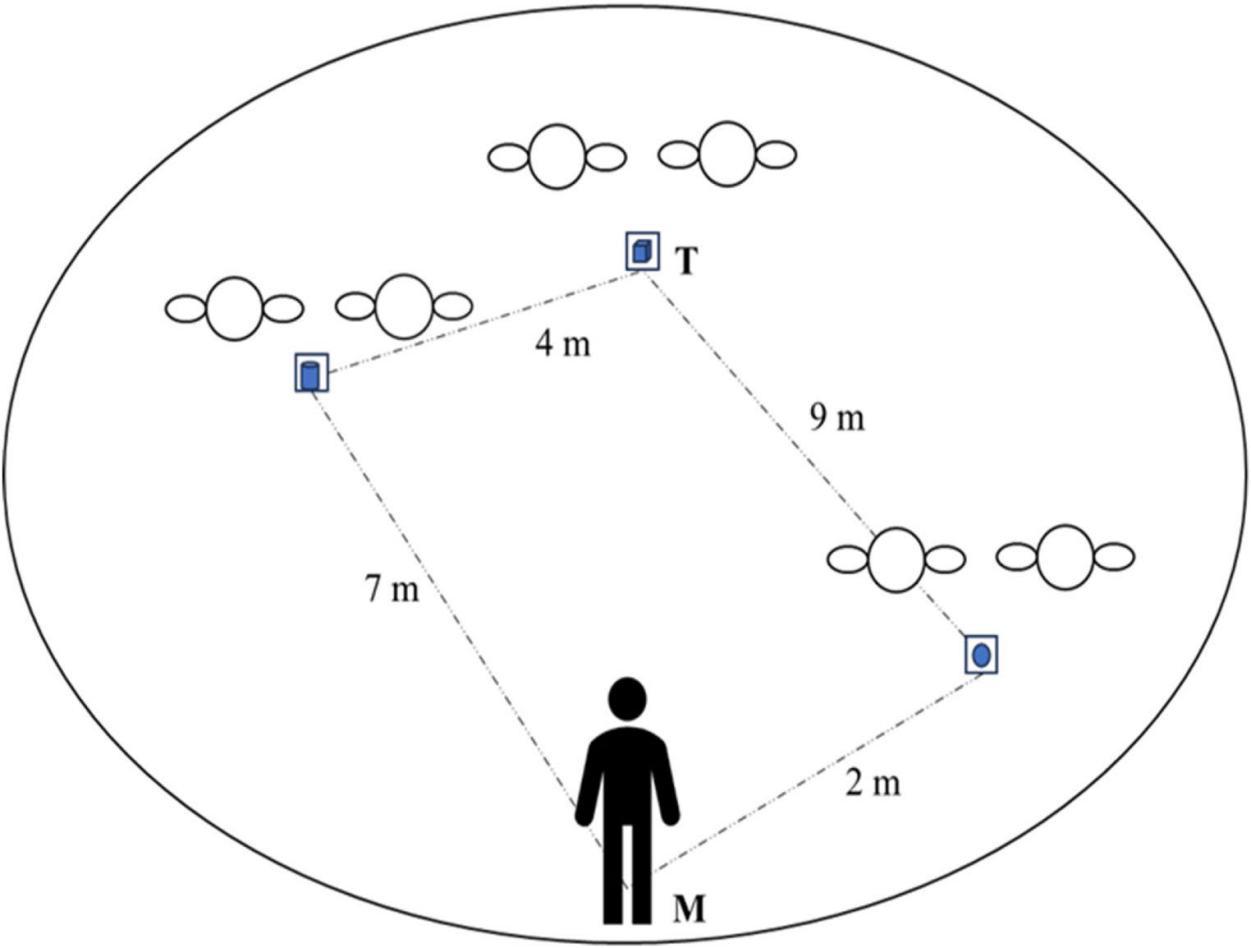
Schematic arrangement of the virtual stimuli. The figure depicts a circular room with three pairs of virtual humans and a triad of geometric stimuli placed in front of each of them. At the bottom of the virtual scenario, there was a mannequin (M) in which participants had to identify themselves to provide relative distance judgments. Black dashed lines indicate inter-object and mannequin-object distances

**Fig. 2:**
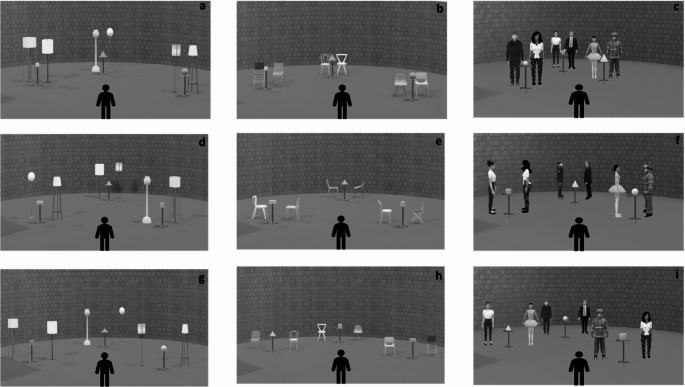
Examples of virtual scenarios in all conditions. From the top left, panels a, d, g depict the lamps (control) condition; at the center panels b, e, h depict the chairs (control) condition; from the top right, panels c, f, i show the virtual humans condition. Moreover, panels c and i show Not-Facing condition while panel f depicts Facing condition. All columns show the interpersonal distances at the Intimate (**a**, **b**, **c**), Personal (**d**, **e**, **f**), and Social (**g**, **h**, **i**) conditions. For the chair and virtual human conditions, the panels also indicate the directionality condition: Facing (**e**, **f**) or Not-Facing (**b**, **h**, **c**, **i**)

Our basic idea is that spatial encoding may be implicitly influenced by the clarity of social information. Social cues can confirm a specific social category, as in the case of two distant people who never look each other in the eyes and are considered strangers (Hall, 1966; Hayduk, 1983; Kendon, 1967; Argyle & Dean, 1965); or can be ambiguous, as in the case of two people who are very close but never look at each other, resulting in uncertainty about their relationship (are they friends or strangers?) (Hall, 1966; Hayduk, 1983; Kendon, 1967; Argyle & Dean, 1965). In the former case, the egocentric encoding should prevail, in line with the spatial memory literature (Iachini, 2024; Ruggiero et al., 2021; Hu et al., 2018; Millar, 1994). In the latter case, instead, when familiar categories do not adequately explain a social context and expectations are not met, individuals should tend to engage in more allocentric (environment-centred) encoding to resolve ambiguities or inconsistencies in the social environment (Arzy & Kaplan, 2022; Fiske & Neuberg, 1990; Hastie et al., 1990). For this reason, we investigated whether the encoding of spatial information according to egocentric and allocentric reference frames could be implicitly affected by social categorizations mechanisms prompted by nonverbal cues. To do that, an explicit spatial memory task was combined with a social (that is virtual humans) vs. non-social (that is chairs and lamps) environment, and two types of non-verbal social information were manipulated: proxemic distances and mutual vs. non-mutual gaze. As the explicit task, a modified Ego-Allo task (Iachini & Ruggiero, 2006; Nunziata et al., 2023; Ruggiero et al., 2014) was used in which participants had to memorize the position of three geometric objects located on small tables and then make egocentric or allocentric judgments. Crucially, each geometric object was in front of a pair of social (i.e., virtual humans) and non-social (i.e., lamps or chairs, as control conditions) stimuli. Each social and non-social pair was located at three different proxemic distances (Hall, 1966): Intimate (45 cm), Personal (75 cm) and Social (150 cm) (see Fig. 1). Virtual humans could have a mutual (Facing condition) or non-mutual (Not-Facing condition) gaze (see Fig. 2). Given the inherent spatial components of both proxemics and gaze, i.e., metric distance the former and directionality the latter, we added two control conditions. Regarding the first factor, to clarify if the implicit proxemic modulation was induced by the social stimuli (i.e., virtual humans) rather than distance “per se”, pairs of virtual humans were compared with pairs of lamps. Furthermore, to clarify the role of directionality, we compared pairs of virtual humans with pairs of chairs (e.g., Gunalp et al., 2019). As with virtual humans, the chairs were shown facing each other or not to resemble the directionality of mutual/non-mutual gaze, respectively (see Fig. 2).

It is interesting to note that with respect to non-verbal cues, Intimate and Social distances generally convey more clearly defined relational meanings. Indeed, Intimate space is typically reserved for close people, such as partners or immediate family members, whereas Social distance is characteristic of formal interactions or interactions between unfamiliar individuals (Hall, 1966; Hayduk, 1983). Therefore, spatial judgements can be efficiently based on egocentric processing, reducing the need for allocentric processing. In contrast, Personal distance constitutes an intermediate interpersonal zone that accommodates a broader range of relational meanings (Hall, 1966; Iachini et al., 2014a, b). This space is highly sensitive to socio-emotional modulation: variations in affect, expectations, or context can shift the preferred comfort distance within the typical 50–140 cm range (Iachini et al., 2014a, b, 2015; Patanè et al., 2017; Ruggiero et al., 2017). Because its relational meaning can support multiple plausible interpretations, observers should rely more on allocentric processing to resolve ambiguity by focusing on the relations between individuals in the environment (e.g., Arzy & Kaplan, 2022; de Vignemont, 2008; Iachini, 2024). As a result, allocentric performance at Personal distance should improve. On the other hand, mutual gaze cue (directionality), being a communicative social cue, may play a role in the understanding of the social environment.

The general hypothesis was that social mechanisms, driven by proxemic distance and gaze direction, should implicitly influence spatial representations according to egocentric and allocentric reference systems. Therefore, in the presence of virtual humans, when the social context is clear (i.e., at intimate and social distances), egocentric performances should be more accurate than allocentric ones; conversely, when the social context is ambiguous (i.e., at personal distance), this should not happen due to higher allocentric accuracy. Finally, with non-social stimuli, we expected a better egocentric than allocentric performance.

Lastly, an important aspect of our social life is the empathic disposition toward others (Davis, 1980). A link between empathy and spatial proximity has been suggested, even with virtual characters (Iachini et al., 2016; Ruggiero et al., 2017; see also Erle & Topolinski, 2015; Nunziata et al., 2025; Nunziata et al., 2022). Given this, we used the Interpersonal Reactivity Index (Davis, 1980) to examine whether spatial behavior was associated with individual empathic disposition.

## Method

### Participants

Forty-two participants (23 females) aged 19–30 (M = 23; SD = 2.73) were recruited in exchange of course credit at the University of Campania “Luigi Vanvitelli” (Italy). Informed consent was obtained from all participants. They had normal or corrected to normal vision and no history of major traumatic injury, drug abuse or neurological disorder. The sample size was determined by means of an a priori power analysis using G*Power, version 3.1.9.4 (Faul et al., 2009) with the following parameters: Cohen’s effect size d = 0.80, α = 0.05, Power (1 − β) = 0.85 (Perugini et al., 2018). The resulting sample size was of 37 participants. The parameters for the analysis were chosen based on a pilot study with a paradigm similar to that of the present study (Nunziata et al., 2023). Recruitment and testing were in conformity with the requirements of the local Institutional Ethics Committee [03/2023]. As an inclusion criterion, we used a distance estimation task to assess the perceptual discrimination capacity. All participants achieved a high accuracy (at least 81%) and none was excluded from the analysis. We adopted a conservative screening-task performance cut‐off (higher > 75%) on our distance‐estimation task to ensure a high level of perceptual discrimination (e.g., National Research Council, 1985). In this way, we could reduce the risk that differences in the task simply reflected perceptual discrimination difficulties (e.g., Torfs et al., 2014; Loomis & Philbeck, 2008; Fine & Jacobs, 2002).

### Apparatus

The experiment took place in a soundproof room of the Cognitive Science and Immersive Virtual Reality Laboratory (CS-IVR, Department of Psychology, University of Campania “L. Vanvitelli”, Italy). The equipment included the OpenSesame software version 3.3 (Mathôt et al., 2012), used on a computer with a 20-inch screen.

#### Experimental setting and stimuli

We replicated the stimuli and spatial arrays as in the Ego-Allo task used in several studies (e.g., Iachini & Ruggiero, 2006; Iachini et al., 2014a, b) within a semicircular virtual room with gray brick walls and a green floor. The stimuli included six easily named real geometric objects, such as Pyramid, Parallelepiped, Cone, Cube, Sphere and Cylinder (Fig. 2). They varied in shades of grey (light = 25%, medium = 50% and dark = 75%), and sizes, large objects, 8 cm x 8 cm (except parallelepiped and cylinder: 8 cm 11 cm) and small, 6 cm x 6 cm (except parallelepiped and cylinder: 6 cm 9 cm). By combining these characteristics, 36 triads were obtained. We used 3D stimuli presented on a screen that depicted reliable three-dimensional spatial cues, such as perspective, depth, and relative size (e.g., Loomis & Knapp, 2003). These cues are known to engage spatial cognitive mechanisms similar to those engaged in the real world (Ruggiero et al., 2014; Ruotolo et al., 2015). Each object was placed on a small table having the following dimensions: width 34 × 34 virtual cm; height 75 virtual cm. The triads were arranged according to the following criteria: (i) the distances between the objects were clearly perceived; (ii) the metric distances were established in such a way that the degree of metrical difficulty was the same for egocentric and allocentric judgments (see Fig. 1a and b; Iachini et al., 2014a, b); (iii) each triad was placed on the circular room corresponding to a mannequin’s midsagittal plane. Indeed, to prevent the subject from adopting uncontrolled egocentric reference points, we explicitly provided an egocentric reference point, in line with other studies (Baess et al., 2018; Heydrich et al., 2013). To this end, a mannequin (M) was adopted within the virtual environment to provide participants with a stable and consistent body reference with which to identify and anchor their spatial judgements. To facilitate the identification process, we did as follows: (i) the mannequin was presented from a natural egocentric perspective; (ii) the midline of the mannequin’s body corresponded to that of the subject; (iii) the subject was explicitly asked to identify with the mannequin. A schematic example of one triad is illustrated in Fig. 1 to show how metric distances were established to ensure the same degree of metrical difficulty for egocentric and allocentric judgments. In this example, the cube (T) was the allocentric reference object. The allocentric distances were the following: cylinder – cube = 4 m, sphere - cube = 9 m. As regards egocentric distances, the sphere and the cylinder were respectively 2 m and 7 m far from the midsagittal plane of the mannequin. The metric difference between the two objects closest to the mannequin (9 − 4) and to the cube (7–2) was the same, i.e., 5 m. The configurations were created using SketchUp© 2019 software.

Behind each object of a triad, there could be either a pair of humans (social stimuli) or a pair of lamps or chairs (non-social stimuli, as controls). Six virtual humans (three females) were selected among a colony of highly realistic Virtual humans (considering the stimuli used in Chen and McNamara’s 2011 study, downloaded from 3D Warehouse - SketchUp). They represented male (height = 175 cm) and female (height = 165 cm) adults aged about thirty years (see Fig. 2). The six virtual humans were combined in such a way as to obtain different pairs.

Regarding the control conditions, the lamps had the same height as male and female virtual humans (mean height = 170 cm), while the chairs measured 90 cm (Gunalp et al., 2019; Nunziata et al., 2023).

Pairs of virtual humans, lamps and chairs could be placed at 45 cm, 75 cm and 150 cm apart, representing the Intimate, Personal and Social proxemic distances, respectively. Each pair of virtual humans and chairs was presented in two conditions: facing each other or not (Not-Facing). Lamps had no directional cue (see Fig. 2).

#### The interpersonal reactivity index

Based on a 5-step Likert-type scale (from 0= ‘‘never true’’ to 5= ‘‘always true’’), the Interpersonal Reactivity Index (IRI; Davis, 1980) measures various facets of dispositional empathy through four subscales (7-items each): *Perspective Taking*, tendency to adopt the psychological point of view of others (e.g., ‘‘I sometimes try to understand my friends better by imagining how things look from their perspective’’); *Fantasy*, tendency to identify with a fictional character (e.g., ‘‘After seeing a play or movie, I have felt as though I was one of the characters’’); *Empathic Concern*, tendency to experience feelings of sympathy and compassion for unfortunate others (e.g., ‘‘I often have tender, concerned feelings for people less fortunate than me’’); *Personal Distress*, tendency to experience discomfort in distress situations (e.g., ‘‘Being in a tense emotional situation scares me’’).

### Procedure

Participants sat in front of a desk on which a computer with a 20-inch screen and a keyboard were placed. After providing written instructions, participants were presented with each geometric object (e.g., cone, cube, etc.) and asked to name it to avoid possible naming problems. Subsequently, there was the metric distance judgment task to assess the ability to discriminate metrics in a virtual space. Participants had to indicate the distance (in meters) of an object that could appear at different positions in relation to a mannequin. Next, a training session began to make participants familiar with the entire procedure. First, the experimenter made sure that the participant’s body midline corresponded to the mannequin’s body midline and instructed the participant to identify with the mannequin. During the training phase, the same setting as during the experimental phase but different stimuli were used. Three objects were presented, each one in front of a pair of non-social stimuli (e.g., coat rack -umbrella). Participants were instructed to memorize the position of the three geometric objects as accurately as possible and then decide whether a given object was the closest/farthest from them or from another object. Once the training phase was completed and the task was clear, the main experiment began.

#### Learning phase

**Fig. 3:**
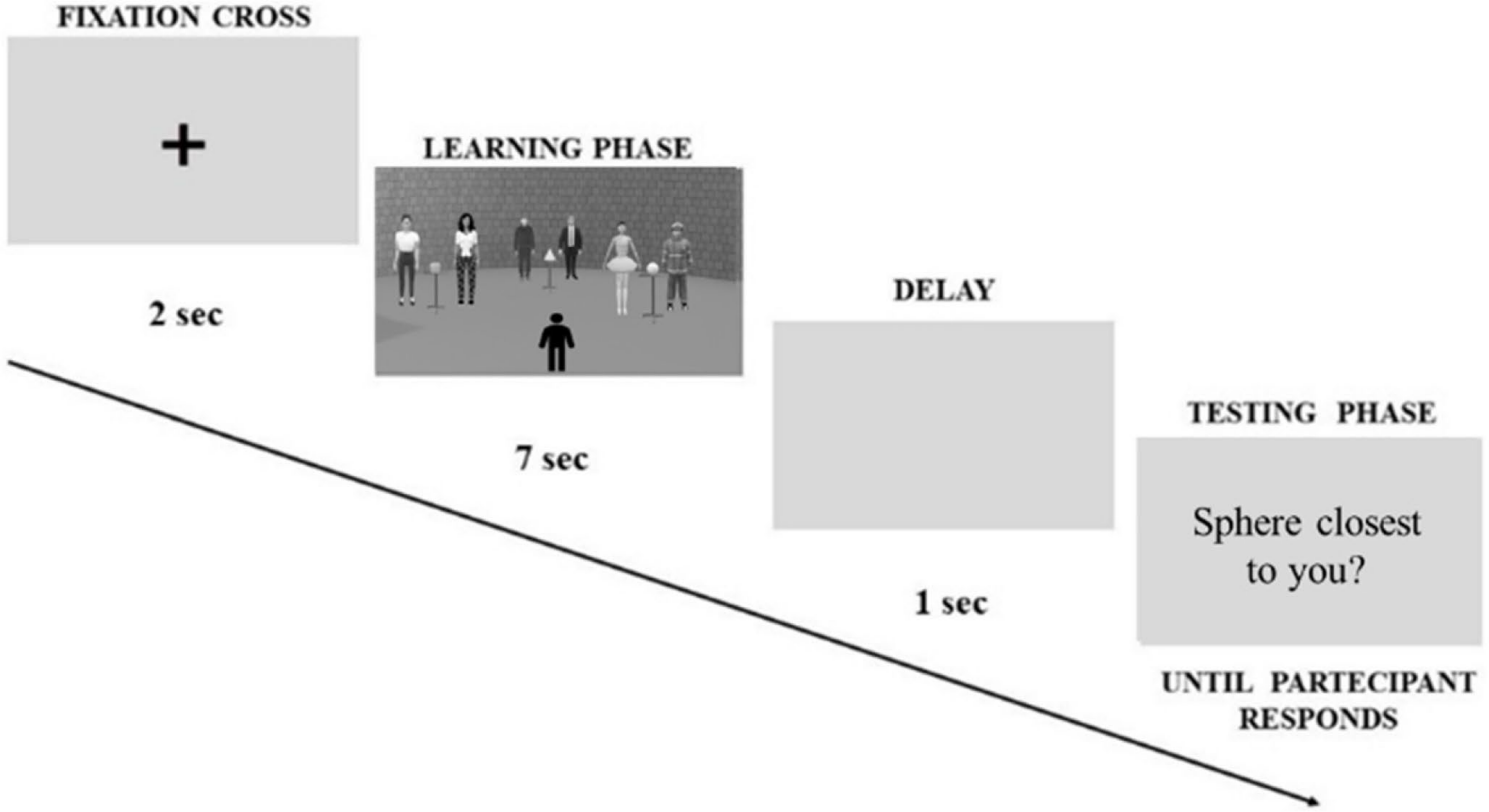
Example of the experimental flow of the Ego-Allo task. Participants saw a fixation cross (2 s), then a scenario (e.g., virtual humans in Not-Facing condition) appeared (7 s) with a triad of geometric objects to be memorized, and after a delay (1 s), they answered. An example of an egocentric question “Sphere closest to you?” is shown in the image; the allocentric question was of the type “Cube closest to the Cone?”

Participants saw a fixation cross for 2 s. Next, participants were instructed to accurately memorize the position of the three geometric objects. No reference was made to the pairs of stimuli (i.e., people, chairs, or lamps) behind the triad. The scenario then disappeared after 7 s, and after a 1-second pause, the test phase began (see Fig. 3).

#### Testing phase

Participants were asked to provide egocentric and allocentric spatial judgments of relative distances about memorised geometric objects by pressing two keys on the keyboard (i.e., L = Yes; K = No) without time limits. The keys were counterbalanced among the participants. The egocentric questions were: “was object X the closest/farthest from you?”; the allocentric questions were: “was object X the closest/farthest from object Y?”. Both egocentric and allocentric questions were presented in a short form (e.g., egocentric: “Sphere closest to you?”; allocentric “Cube closest to the cone?”) (Fig. 3). Five blocks were presented randomly: two blocks for the virtual humans (Facing and Not-Facing conditions), each consisting in 18 triads; two blocks for the chairs (Facing and Not-Facing conditions), each consisting in 18 triads each; one block for the lamp condition consisting in 18 triads. For each triad, a single question was asked, either egocentric or allocentric. Accuracy (mean %, 1 = correct; 0 = incorrect) was recorded for each judgment, for a total of 90 judgments (45 egocentric, 45 allocentric). The egocentric and allocentric questions were presented in random order within the experimental blocks. Moreover, the distances between target objects were counterbalanced between different conditions and the order of presentation of the questions was first randomized and then counterbalanced across subjects. Next, participants were asked to complete the IRI questionnaire. The experiment lasted about 40 min.

## Statistical analyses

Outliers of accuracy data were defined as values ± 2.5 standard deviations from the mean of the corresponding condition (Cousineau & Chartier, 2010). For each participant, mean accuracy and standard deviation were calculated separately for each experimental condition. Thirteen data values (1% of the data) were replaced with the average of their corresponding condition. Descriptive analyses showed that skewness and kurtosis values across all variables were normal: average skewness = −0.38 range: −2.11 to 0.26; average kurtosis = − 0.62 range: −1.60 to 2.60 (Chou & Bentler, 1995).

To assess the impact of proxemic distances on frames of reference, a repeated measure ANOVA was carried out with the following factors: 2 (Stimuli: Virtual Humans-Not-Facing vs. Lamps) x 3 (Proxemic distances: Intimate vs. Personal vs. Social) x 2 (Frames of reference: Egocentric vs. Allocentric). To assess the impact of proxemic distances and gaze direction on social and non-social stimuli, a repeated measure ANOVA with four factors was performed: 2 (Stimuli: Virtual Humans vs. Chairs) x 2 (Directionality: Facing vs. Not-Facing) x 3 (Proxemic distances: Intimate vs. Personal vs. Social) x 2 (Frames of reference: Egocentric vs., Allocentric). The Tukey HSD test was used to analyse post-hoc effects and the magnitude of the significant effects was indicated by partial eta squared (η^2^_p_). Finally, a Pearson’s correlation between Virtual Humans Facing and Not-Facing conditions (acc.) and mean scores of the four IRI sub-scales was performed. The False Discovery Rate method (Benjamini & Hochberg, 1995) was used to control for multiple comparisons.

## Results

### Virtual humans (Not-Facing) vs. Lamps

**Fig. 4:**
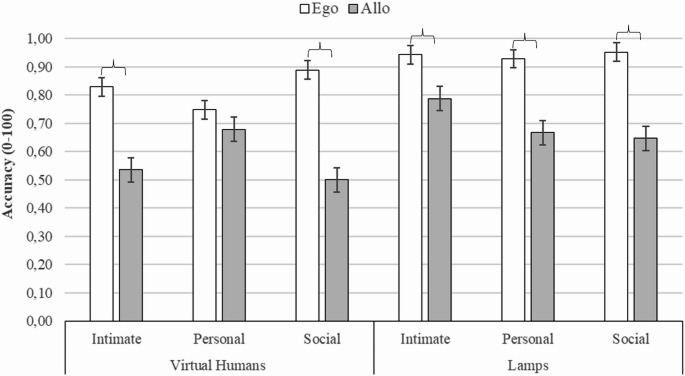
The figure shows mean accuracy of Egocentric and Allocentric judgements as a function of the Social/Non-social stimuli(i.e., Virtual Humans-Not-Facing vs. Lamps) and Proxemic distances (i.e., Intimate vs. Personal vs. Social). Brackets indicate statistically significant differences. Vertical thin bars represent standard error

A main effect of Stimuli, F (1, 41) = 26.917, *p* <.0000006, η^2^_p_ = 0.39, emerged with participants being more accurate in the Lamps condition (M= 0.83; SD=0.23; 95% CI = [0.759; 0.897]) than the Virtual Humans (M=. 70; SD=0.30, 95% CI = [0.607, 0.787]) one. A main effect of Frames of Reference, F (1, 41) = 115.629, *p* <.00000, η^2^_p_ = 0.73, was also found due to participants being more accurate in the Egocentric (M= 0.88; SD=0.18, 95% CI = [0.825, 0.936]) than Allocentric (M= 0.64; SD=0.30, 95% CI = [0.555, 0.734]) judgments. An interaction between Proxemic distances and Frames of References appeared: F (2, 82) = 9.554, *p* <.0001, η^2^_p_ = 0.18. Post-hoc test revealed an advantage of Egocentric over Allocentric judgments in all proxemic distances (at least *p* <.0001). Moreover, Allocentric judgments were more accurate at Personal than Social distance (*p* =.01). Finally, a three-way interaction between Proxemic distances, Frames of References and Social/Non-social stimuli was revealed: F (2, 82) = 8.250, *p* <.0005, η^2^_p_ = 0.16 (see Fig. 4). The results indicated that only social stimuli affected the use of reference frames, as shown by the improvement of Allocentric performance with social stimuli at Personal distance. Specifically, in the Virtual Humans at Intimate and Social distances, Egocentric judgements were more accurate than Allocentric ones (*p* <.0001). In contrast, at Personal distance there was no significant difference between the two reference systems (*p* >.05). As for Virtual Humans, Egocentric judgements at Social distance were more accurate than Egocentric judgements at Personal distance (*p* =.03), while Allocentric judgments were more accurate at Personal than Intimate and Social distances (at least *p*<.03). Regarding the Lamps condition, an Egocentric over Allocentric advantage was found in all proxemic distances (at least *p*<.02). Moreover, Allocentric judgements at Intimate and Social distances were more accurate with Lamps than Virtual Humans (at least *p*<.001), while no difference emerged at Personal distance (*p* >.05). Finally, Egocentric judgments were more accurate at Intimate, Personal and Social distances in the Lamps condition than at Personal distance in the Virtual humans one (at least *p*<.001).

### Proxemics and directionality: virtual humans vs. Chairs

A main effect of Stimuli, F (1, 41) = 29.609, *p* < 000003, η^2^_p_ = 0.42, was revealed. Participants were more accurate in the Virtual Humans (M= 0.69; SD=0.28; 95% CI = [0.609, 0.780]) than Chairs (M= 0.61; SD=0.29; 95% CI = [0.521, 0.698]) condition. A main effect of Frames of References, F (1, 31) = 51.704, *p*<.000000, η^2^_p_ = 0.59, showed that Egocentric judgments (M= 0.72; SD=0.27; 95% CI = [0.634, 0.797]) were more accurate than Allocentric ones (M= 0.59; SD=0.30; 95% CI = [0.499, 0.679]).

A two-way Social/Non-social stimuli x Proxemic distances interaction appeared, F (2, 82) = 21.193, *p* <.000000, η^2^_p_ = 0.34). The post hoc test showed that spatial performance with Virtual Humans at Personal distance had an advantage over all other combinations (at least, *p* <.01). In the Chairs condition, Intimate and Social distances were more accurate than Personal ones (at least *p*<.0002). Moreover, a two-way Social/Non-social stimuli x Frames of References interaction emerged F (1, 41) = 6.035, *p* <.01, η^2^_p_ = 0.12): Egocentric judgments with Virtual Humans were more accurate than all other conditions (*p* <.0002). In the Chair condition, Egocentric judgments were more accurate than Allocentric ones (*p* <.01). Furthermore, a two-way Proxemic distances x Frames of References interaction appeared, F (2, 82) = 4.237, *p* <.01, η^2^_p_ = 0.09). In all proxemic distances, Egocentric judgments were more accurate than Allocentric ones (at least *p* < 03).

**Fig. 5:**
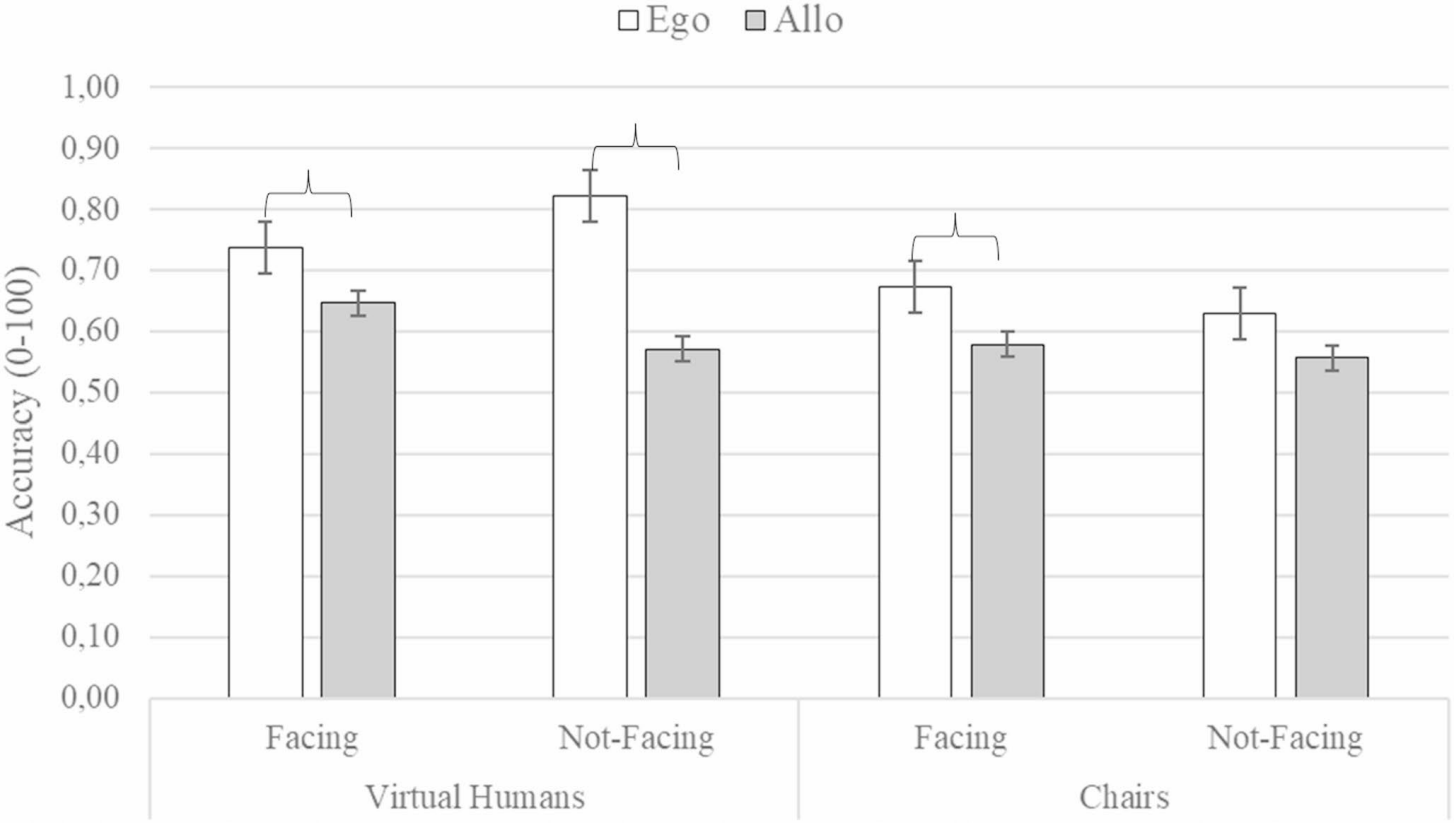
The figure shows mean accuracy of Egocentric and Allocentric judgements as a function of the Social/Non-social stimuli (i.e., Virtual Humans vs. Chairs) and Directionality (i.e., Facing vs. Not-Facing). Brackets indicate statistically significant differences. Vertical thin bars represent standard error

A three-way interaction between Stimuli, Directionality and Frames of References was found, (F (2, 82) = 10.584, *p* <.002, η^2^_p_ = 0.21) (see Fig. 5). In the Facing condition with Virtual Humans, Egocentric judgments were more accurate than all Allocentric judgments (at least *p*<.03) and they were also more accurate than Egocentric Judgments with Chairs in Not-Facing condition (*p*=.007). Additionally, Allocentric judgments were more accurate with Facing Virtual Humans than Not-Facing Chairs (*p*<.05). In the Not-Facing condition with Virtual Humans, Egocentric judgments had an advantage over all other conditions (at least *p*<.0002) and they also tended to be more accurate than Egocentric judgments with Virtual Humans in the Facing condition (*p* =.07).

**Fig. 6:**
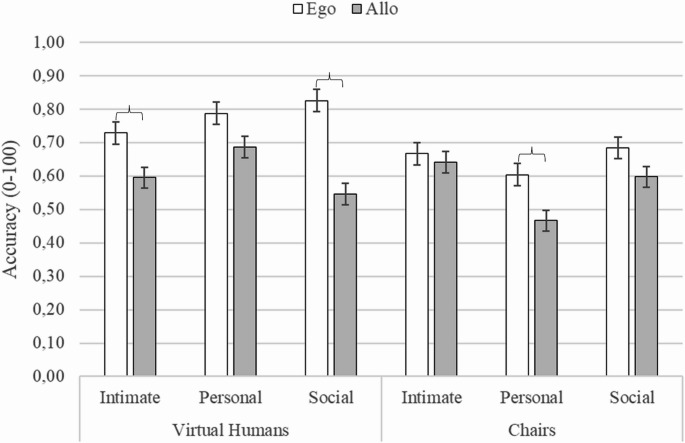
The figure shows mean accuracy of Egocentric and Allocentric judgements as a function of the Social/Non-social stimuli (i.e., Virtual Humans vs. Chairs) and Proxemic distances (i.e., Intimate vs. Personal vs. Social). Brackets indicate statistically significant differences. Vertical thin bars represent standard error

A three-way interaction between Stimuli, Proxemic distances and Frames of References was found, (F (2, 82) = 6.105, *p* <.003, η^2^_p_ = 0.13) (see Fig. 6). Key differences highlighted by post-hoc analyses showed that the presence of Virtual Humans enhanced Egocentric spatial judgments at Intimate and Social distances, while Allocentric accuracy improved mainly at Personal distance. Indeed, with Virtual Humans, Egocentric judgments at Intimate and Personal distances were more accurate than Allocentric ones at Intimate and Social distances (at least *p* <.007). Moreover, Egocentric judgments at Intimate distance were more accurate than Egocentric judgments with Chairs at Personal distance (*p*=.01), and more accurate than Allocentric judgments with Chairs at Personal and Social distances (at least *p* <.008). Similarly, Egocentric judgments at Personal distance were more accurate than Allocentric judgments with Chairs at all distances (at least *p* <.001), and more accurate than Egocentric judgments with Chairs at Personal distance (*p* =.0001). Furthermore, Egocentric judgments at Social distance were more accurate than Allocentric judgments at all proxemic distances (at least *p* <.003) and more accurate than all Chairs conditions (at least *p* <.003). As regards Allocentric judgments with Virtual Humans, they were more accurate at Personal than Social distances (*p* =.003), and more accurate than Allocentric judgments with Chairs at Personal distances (*p* =.0001). In the presence of Chairs, at Personal distance, Egocentric judgments were more accurate than Allocentric ones (*p* =.004), and both spatial judgments at Intimate and Social distances were more accurate than Allocentric Judgments at Personal distances (at least *p* <.004).

**Fig. 7:**
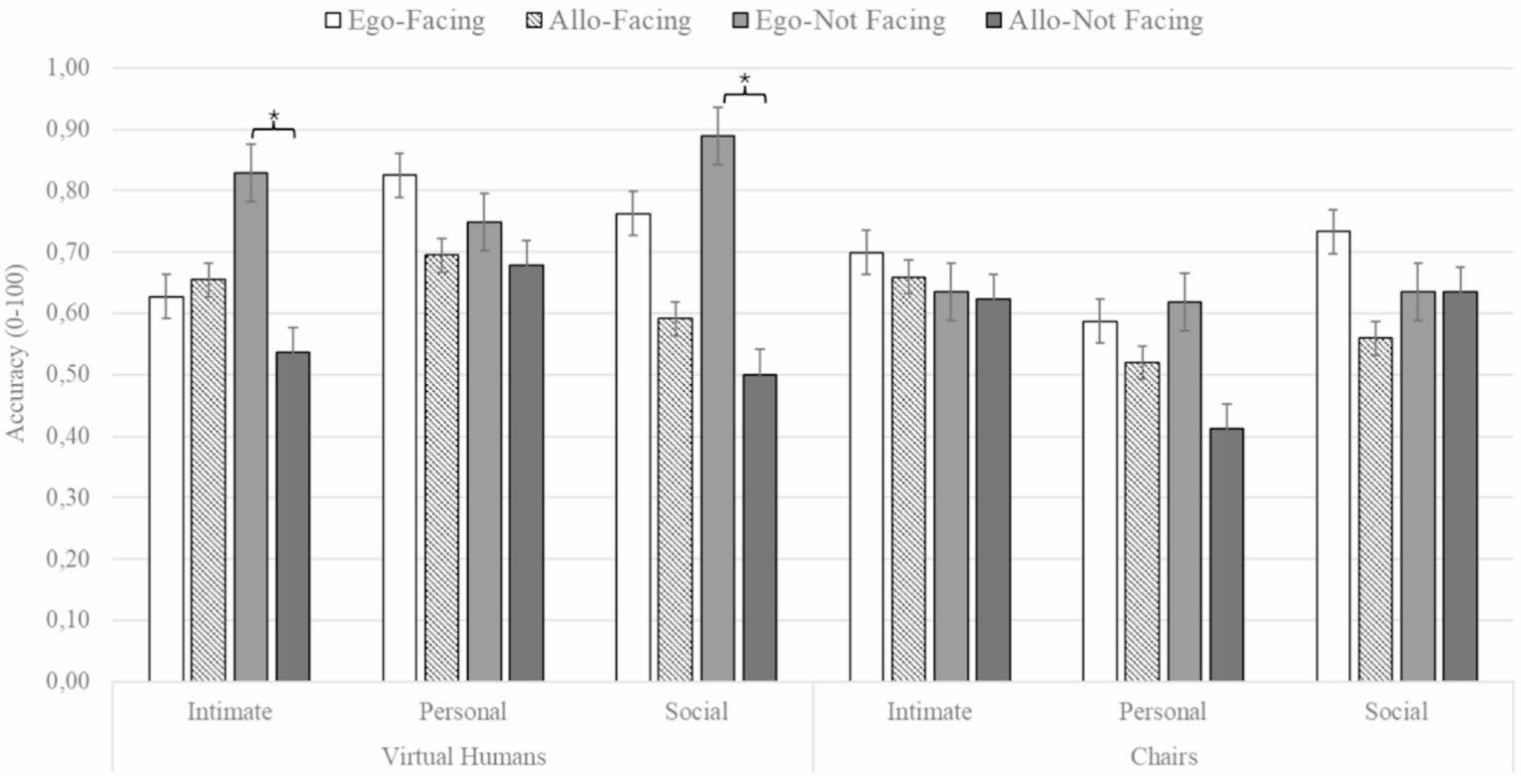
The figure shows the mean accuracy of egocentric and allocentric judgments as a function of stimuli (virtual human or chair), proxemic distances (Intimate or Personal or Social), and directionality (Facing or Not-Facing). Brackets indicate statistically significant differences. Thin vertical bars represent standard error

Finally, a four-way interaction between Stimuli, Proxemic distances, Directionality and Frames of References emerged (F (2, 82) = 7.097, *p* <.001, η^2^_p_ = 0.14) (see Fig. 7). The key post-hoc comparisons indicated that proxemic distance interacted with directionality in affecting spatial judgments: in Not-Facing conditions, Egocentric judgments were more accurate at Intimate and Social distances while the allocentric performance improved at Personal distance; instead, in Facing conditions, the Egocentric advantage was mitigated. Indeed, considering the Virtual Humans Not-Facing condition, Egocentric judgments at Intimate distance were more accurate than Allocentric ones (*p* =.002), whereas at Social distance they were more accurate than Allocentric ones at Social and Intimate distances in both Facing and Not-Facing conditions (at least *p* <.05), and more accurate than Egocentric Judgments in the Intimate Facing condition (*p* =.01). On contrast, at Personal distance there was no significant difference between spatial judgments. Furthermore, in the Virtual Humans Not-Facing condition at Social distance, Egocentric judgments were more accurate than all Allocentric ones with Chairs in both Not-Facing and Facing conditions (at least *p* <.05), and than Egocentric judgments with Chairs Not-Facing and Facing conditions at Personal distances (at least *p* <.01). Considering the Virtual Humans Facing condition, the comparison between Egocentric and Allocentric judgments did not reach significance. At Social distance, Egocentric judgments in the Facing condition were more accurate than Allocentric ones in Not-Facing conditions (at least *p*<.05) and tend to be more accurate than Allocentric judgments in the Virtual Humans Not-Facing condition at Intimate distance (*p*=.06). Moreover, in the Virtual Humans Facing condition at Personal distances, Egocentric judgments were more accurate than Allocentric ones in the Chairs Facing condition at Personal and Social distances (at least *p*<.001) and more accurate than Egocentric judgments in the Chairs Facing condition at Personal distances (*p*=.04). Finally, with Virtual Humans in both Facing and Not-Facing conditions at Personal distance, all Egocentric and Allocentric judgments were more accurate than Allocentric judgments in the Chair Not-Facing condition at Personal distance (at least *p*<.05).

### Correlation analysis

A positive correlation (*p* =.000, *r* =.56) between the accuracy of Egocentric judgments in the Personal Not-Facing condition and the Fantasy subscale was found: the more participants tended to identify with fictional characters, the more accurate the Egocentric judgments were. Moreover, a positive correlation between the accuracy of Egocentric judgments in the Facing Social distances and the Fantasy (*p* =.000; *r* =.55), Empathic Concern (*p* =.000; *r* =.70) and Perspective taking (*p* =.000; *r*=.64) subscales was found: the more participants tended to empathize with others, the higher Egocentric accuracy. A positive correlation (*p* =.002, *r* =.46) between the accuracy of Allocentric judgments in the Intimate Facing condition and the Empathic Concern subscale score was found: the more they were able to empathize and had feelings of concern for others who were unfortunate, the more accurate the Allocentric judgments were. Finally, a positive correlation (*p* =.004, *r* =.44) between the accuracy of Allocentric judgments in the Social Facing condition and the Personal Distress subscale score was found: the more prone people were to anxiety and personal distress in tense interpersonal contexts, the more accurate allocentric judgments were.

## Discussion

When we are in a new place, we encode spatial information according to egocentric (subject-object) and allocentric (object-object) reference frames. However, the environment is characterized not only by “objects” but also by people around us (Richardson & Gobel, 2015; Gobel et al., 2018; Farrow et al., 2011), and social information can have a relevant impact on our spatial memory.

In this paper, we asked whether proxemic distance and gaze direction, i.e., non-verbal cues that mediate the individuation of social categories (McGarty et al., 2015), could implicitly influence how we represent surrounding spatial objects according to frames of reference. Participants were asked to memorize triads of geometric stimuli that could appear along with pairs of social (i.e., virtual humans with mutual/non-mutual gaze) or non-social (i.e., lamps or chairs, as control conditions) stimuli.

Overall, the results provide significant support for our hypotheses, demonstrating that non-verbal social signals (i.e., proxemic distance and gaze direction) affect the processing of spatial reference frames of contextual stimuli.

Consistent with a long-standing body of literature, egocentric spatial judgments were generally more accurate than allocentric ones (O’Keefe & Nadel, 1978; Paillard, 1991; Ruggiero et al., 2014; Iachini & Ruggiero, 2006). This advantage was evident in all our control conditions (with the pairs of lamps and chairs) and in social situations with virtual humans that did not require effort in clarifying social relationships. Regarding the stimuli, performance was better with virtual humans than with chairs (in both Facing and Not-Facing conditions), but not better than with lamps. The lamps had low visual complexity and low social characterization, which likely helped isolate the role of mere spatial distance from social nuances in the processing.

A key finding was the modulation of this egocentric advantage with proxemics. When the only social information available was proxemic distance (i.e., in the virtual humans Not-Facing condition), the egocentric encoding was dominant at both the Intimate and Social distances, showing the typical egocentric advantage over allocentric processing (e.g., Golledge, 1992; Hart & Moore, 1973; Levelt, 1989; Piaget & Inhelder, 1967; Shelton & McNamara, 1997). These proxemic distances offer non-verbal cues that are straightforward to interpret, facilitating immediate social categorization and leaving cognitive resources available for spatial processing (Frith & Frith, 2007; de Vignemont, 2008). Conversely, at Personal distance (i.e., physical dimension where friends and even informal interactions can take place; Hall, 1966; Hayduk, 1983; Iachini et al., 2014a, b), the egocentric advantage disappeared, with allocentric performance nearly matching egocentric performance. Consistently, allocentric judgments with virtual humans were more accurate at Personal distances than at Intimate or Social distances. Allocentric improvement could be linked to further environment-centred processes to clarify the social structure and mutual relationships of virtual humans (de Vignemont, 2008; Taylor & Tversky, 1996; McGarty et al., 2015). This is further corroborated by comparisons with non-social stimuli: allocentric judgments at Personal distances were more accurate with virtual humans than with chairs.

It is interesting to note that, for Chairs in the Not-Facing condition, the pattern is opposite to that observed with virtual humans: at Intimate and Social distances, no difference between reference frames emerged, whereas at Personal distance an egocentric advantage was found, with the allocentric system being less accurate than all other judgments. Although these stimuli were used to check the role of directionality, the results deserve a comment. As demonstrated by Faur and Laursen (2022) and Greenberg (1976), chairs can be considered objects with social affordances capable of implicitly evoking human interactions in typical contexts of use (e.g., classrooms, offices, meetings). Previous studies have shown that seating arrangements at Personal distances are associated with greater opportunities for friendly social interactions and activate implicit representations of interpersonal relationships as potential ‘places of interaction’ (Faur & Laursen, 2022). Speculatively, this may have made it easier to detect the implicit social meaning, thus giving rise to the usual egocentric advantage over allocentric performance. As regards Intimate and Social distances, instead, it is possible that the absence of clear social cues (as those provided by virtual humans) required deeper engagement of allocentric processing, resulting in similar accuracy for both reference frames. However, this interpretation should be viewed with caution pending further studies.

The introduction of gaze direction further clarified these mechanisms. In the pairs of virtual humans with a mutual gaze, the egocentric judgments were more accurate than all allocentric judgments with both virtual humans and chairs. In the presence of virtual humans not gazing at each other, the egocentric judgments were not only more accurate than all allocentric judgments but also than egocentric judgments in the presence of chairs. A crucial aspect emerges when considering the various factors together (stimuli, directionality, proxemic distances and reference systems). Indeed, the results suggest that the proxemics combined with the directionality of gaze may give rise to a more complex picture. The performance with virtual humans in Not-Facing conditions confirmed the pattern obtained in the comparison with the lamps: the egocentric processing had an advantage over the allocentric one at Intimate and Social distances, while no difference emerged between the systems at Personal distance. In contrast, in the Facing condition, although egocentric performance was more accurate at personal and social distances (see Fig. 7), the comparison with allocentric performance did not reach statistical significance. Our findings suggest that gaze, as a powerful communicative signal (Kleinke, 1986; Driver et al., 1999; Batki et al., 2000; Emery, 2000), when combined with proxemic distance, increases the demand to interpret the social meaning of the relational structure. Indeed, mutual gaze may convey a range of different social interactions, such as affiliative, neutral, or antagonistic ones, which require a more in-depth social interpretation. While this process contributes to clarifying social meaning, it simultaneously increases its complexity. Instead, the Not-Facing conditions revealed more clearly the effect of proxemic distance. However, more studies are needed to disentangle the weight of the various factors.

Similarly, in the presence of chairs and independent of the other factors (proxemics and directionality), no difference emerged between the frames of reference. Although introduced as non-social stimuli, as mentioned above, chairs seem to activate an implicit representation of the social relationship (Sommer, 1969; Greenberg, 1976). From this perspective, chairs do not evoke the direct intentionality typical of social stimuli such as people (Driver et al., 1999; Batki et al., 2000), but they can still activate relational cognitive schemas related to the potential presence of people. Their ambiguity, neither fully social nor fully neutral, could explain the similar reliance on egocentric and allocentric reference frames.

Importantly, these results show how social and spatial processing are intertwined. Social psychology theories suggest that social perception begins with simple categorization (or stereotyping) and becomes more complex when categories do not immediately fit, requiring more detailed, individuated evaluations (Fiske & Neuberg, 1990; Hastie et al., 1990). From this view, previous work has pointed out that the duality of social interaction and social observation functions parallels the duality of egocentric and allocentric social frames of reference (Frith and de Vignemont, 2005; de Vignemont, 2008). Indeed, social observation should be oriented toward persons in an interaction characterised by immediate social meaning, while social observation should be oriented toward the interaction between persons (other-other) and should require inspection of people interaction outside the viewer (see de Vignemont, 2008).

Our findings suggest that the structure of social information available in the physical environment, even though irrelevant to the main task, induces systematic biases in spatial representation through egocentric and allocentric reference frames. Specifically, the allocentric representation appears more accurate when the ambiguity of external social cues requires further processing of other-other socio-spatial relations, whereas the egocentric representation maintains its usual advantage when the implicit social meaning is clear. These findings support the view that there is a close similarity between social cognitive maps and spatial representations (e.g., de Vignemont, 2008; Peer et al., 2021; Tavares et al., 2015). More specifically, by demonstrating the implicit influence of social factors on spatial processing, they help us understand the mechanisms through which the human mind represents, interprets, and navigates social and physical environments. From this view, the current study contributes to clarifying how spatial representations are intrinsically affected by socially meaningful cues, and how cognitive mechanisms preserve coherence in both physical and social environments.

Finally, the IRI questionnaire suggested that the more one empathizes with the feelings and actions of the fictional characters, the more accurate body-centred judgments were, and the more participants emphatically felt to be oriented toward the others, the more accurate they were in both egocentric and allocentric judgments. This underlies the close relationship between the spatial processing according to reference systems and the capacity to put in the others’ shoes (Iachini & Ruggiero, 2021; Nunziata et al., 2022; Nunziata et al., 2025).

## Conclusion

In conclusion, the processing of social factors, mediated by the categorization of non-verbal behaviours, implicitly affects spatial representation through egocentric and allocentric reference frames (Maddox et al., 2008; Arzy & Kaplan, 2022; Tavares et al., 2015; de Vignemont, 2008; Taylor & Tversky, 1996). When the social context is clear, egocentric performance is more accurate than allocentric one. Instead, when social information cannot be easily assigned to specific relational categories, an advantage of the allocentric system emerges, likely reflecting the need to reduce ambiguity through a knowledge-oriented mechanism directed toward others (social allocentric). Overall, our results highlight that spatial encoding is not a purely spatial process, but one affected by social factors, offering novel insights into the dynamic interplay between social and spatial cognition.

### Limitations

A potential limitation of the present study is the use of three-dimensional stimuli presented on a screen, although they included reliable spatial cues such as perspective, depth, and relative size (e.g., Loomis & Knapp, 2003). Indeed, these cues are known to activate spatial cognitive mechanisms similar to those involved in real-world perception (Ruggiero et al., 2014; Ruotolo et al., 2015); however, the absence of an immersive environment may have reduced, at least in part, the ecological validity of the task.

A further consideration concerns social stimuli, which, despite having produced an effect on spatial encoding, could be refined in future studies through the use of directional cues that are truly socially neutral. This would allow for a clearer distinction between the contribution of purely directional information and that of social information.

## Data Availability

No datasets were generated or analysed during the current study.
